# Effect of chemotherapy on the microbiota and metabolome of human milk, a case report

**DOI:** 10.1186/2049-2618-2-24

**Published:** 2014-07-11

**Authors:** Camilla Urbaniak, Amy McMillan, Michelle Angelini, Gregory B Gloor, Mark Sumarah, Jeremy P Burton, Gregor Reid

**Affiliations:** 1 Lawson Health Research Institute, 268 Grosvenor Street, London, ON N6A 4V2, Canada; 2 Department of Microbiology & Immunology, Western University, London, ON N6A 5C1, Canada; 3 Perinatal and Women’s Health, London Health Sciences Centre, London, ON N6A 4L6, Canada; 4 Department of Biochemistry, Western University, London, ON N6A 5C1, Canada; 5 Department of Chemistry, Western University, London, ON N6A 5C1, Canada; 6 Agriculture and Agri-Food Canada, London, ON N5V 4T3, Canada

**Keywords:** 16S rRNA gene sequencing, Human milk microbiome, Metabolome

## Abstract

**Background:**

Human milk is an important source of bacteria for the developing infant and has been shown to influence the bacterial composition of the neonatal gut, which in turn can affect disease risk later in life. Human milk is also an important source of nutrients, influencing bacterial composition but also directly affecting the host. While recent studies have emphasized the adverse effects of antibiotic therapy on the infant microbiota, the effects of maternal chemotherapy have not been previously studied. Here we report the effects of drug administration on the microbiota and metabolome of human milk.

**Methods:**

Mature milk was collected every two weeks over a four month period from a lactating woman undergoing chemotherapy for Hodgkin’s lymphoma. Mature milk was also collected from healthy lactating women for comparison. Microbial profiles were analyzed by 16S sequencing and the metabolome by gas chromatography–mass spectrometry.

**Findings:**

Chemotherapy caused a significant deviation from a healthy microbial and metabolomic profile, with depletion of genera *Bifidobacterium*, *Eubacterium*, *Staphylococcus* and *Cloacibacterium* in favor of *Acinetobacter*, *Xanthomonadaceae* and *Stenotrophomonas*. The metabolites docosahexaenoic acid and inositol known for their beneficial effects were also decreased.

**Conclusion:**

With milk contents being critical for shaping infant immunity and development, consideration needs to be given to the impact of drugs administered to the mother and the long-term potential consequences for the health of the infant.

## Background

Colonization of the neonatal gut plays a pivotal role in gastrointestinal, immunological and neurological development, with one of the initial sources of bacteria being the mother’s milk [1,2]. Breastfed infants have been shown to have lower incidences of asthma, diarrhea, and necrotizing enterocolitis compared with formula-fed infants [3]. This protective effect may be due, in part, to the types of bacteria present in milk, as infants fed formula supplemented with probiotics were better protected against these conditions compared to those just fed formula [4-6]. The bacteria acquired during infancy can influence disease risk later in life and play a major role in determining the future composition of the adult microbiome [5]. Thus, factors that affect the milk microbiota have important health consequences for the child not only during development but also into adulthood. In addition to the microbiota, the metabolites of human milk, such as fatty acids, carbohydrates, proteins and vitamins, also play an important role in infant development and long-term health [7-10].

**Figure 1:**
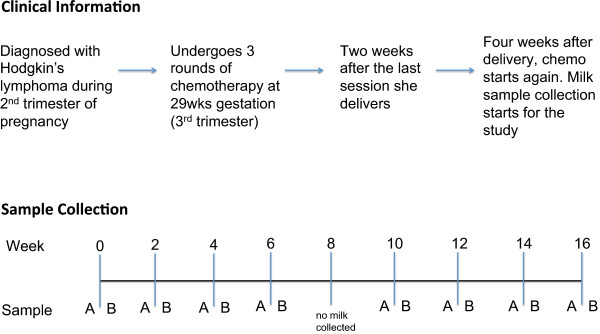
**Summary of clinical data and sample collection.** Milk samples were collected from a lactating woman undergoing chemotherapy for Hodgkin’s lymphoma. Milk samples were collected every 2 weeks over a 4-month period. At each session milk was collected 15 to 30 minutes before (sample A) and after (sample B) chemotherapy. The duration of chemotherapy treatment was 2 hours. No milk was collected at week 8 due to scheduling conflicts.

Post-delivery, many women are prescribed pharmaceutical agents for various reasons. While most over-the-counter drugs and antibiotics are not contraindicated during breast feeding [11,12], when it comes to chemotherapeutics, the recommendation is that breastfeeding should be avoided until the drug has been cleared from the milk [13]. In a case report of a 70 mg infusion of cisplatin, no detectable levels were found in milk after 66 hours [14], and in another case study using doxorubicin (trade name Adriamycin), no detectable levels were seen after 72 hours [15]. In our particular study, the subject was advised that breastfeeding could resume 12 days after each chemotherapy session.Here we present the first report on the effects of chemotherapy on microbial and metabolomic profiles in human milk over a 4-month period in a breastfeeding woman undergoing treatment for Hodgkin’s lymphoma (Figure 1).

## Methods

### Clinical samples and study design

Ethical approval was obtained from Western Research Ethics Board and Lawson Health Research Institute, London, Ontario, Canada. Subjects provided written consent for sample collection and subsequent analyses.

### Milk collection and processing

Mature milk was collected from a lactating woman undergoing the ABVD chemotherapy regime (Adriamycin (40 mg), Bleomycin (16 units), Vinblastine (9.6 mg), Dacarbazine (600 mg) for Hodgkin’s lymphoma at the London Health Sciences Center, London, Ontario. Mature milk was also collected from 8 healthy women recruited from London, Ontario and the surrounding area. Wearing sterile gloves the women cleaned their nipple and surrounding area with sterile saline swabs to reduce the presence of skin bacteria. Milk was collected using a sterile HygieniKit Milk Collection System (Ameda, Buffalo Grove, IL, USA) attached to an electric breast pump. Between 5 and 15 ml of milk was pumped into a sterile tube and kept on ice until transfer to the laboratory, which occurred within 1 hour of collection. Samples were aliquoted and stored at -20°C until DNA extraction.

### DNA isolation

After thawing on ice, 2 ml of milk were spun down at 13,000 *g* for 10 minutes and the supernatant discarded. The pellet was then homogenized in 1.4 ml of ASL buffer (QIAamp® DNA Stool Kit, QIAGEN: Valencia, CA, USA) and 400 mg of 0.1 mm diameter zirconium-glass beads (BioSpec Products, Bartlesville, OK, USA). Mechanical and chemical lyses were performed by bead beading at 4,800 rpm for 60 s, then 60 s on ice (repeated twice) using a mini-beadbeater-1 (BioSpec Products) and then incubated at 95°C for 5 minutes. Subsequent procedures were performed using the QIAGEN QIAamp® DNA Stool Kit according to the manufacturer’s protocol, with the exception of the last step in which the column was eluted with 120 μl of elution buffer. DNA was stored at -20°C until further use.

### V6 16S rRNA gene sequencing

#### PCR amplification

The genomic DNA isolated from the clinical samples was amplified using the barcoded primers V6-LT: 5′CCATCTCATCCCTGCGTGTCTCCGACTCAGNNNNNCWACGCGARGAACCTTACC3′ and V6-RT: 5′ CCTCTCTATGGGCAGTCGGTGATACRACACGAGCTGACGAC3′, which amplify the V6 hypervariable region of the 16S rRNA gene. The PCR was carried out in a 40 μl reaction containing 5 μl of DNA template (or nuclease-free water as a negative control), 1.5 mM MgCl_2_, 0.8 μM of each primer, 4 μl of 10× PCR Buffer (Invitrogen, Burlington, ON, Canada), 0.2 mM dNTPs, 0.05U *Taq* Polymerase (Invitrogen) and 0.15 μg/μl bovine serum albumin. Thermal cycling was carried out in an Eppendorf Mastercyler under the following conditions: initial denaturation at 95°C for 2 minutes followed by 25 cycles of 95°C for 1 minute, 55°C for 1 minute and 72°C for 1 minute. After amplification, the DNA concentration was measured with the Qubit® 2.0 Fluorometer (Invitrogen) using the broad range assay. Equimolar amounts of each PCR product were pooled together and purified using the QIAquick PCR purification kit (QIAGEN). The PCR purified sample was then sent to the London Regional Genomics Center, London, Ontario, Canada for V6 16S rRNA gene sequencing using the Ion Torrent platform as per the center’s standard operating procedure.

#### Sequence processing and taxonomic assignment

Custom Perl and Bash scripts were used to de-multiplex the reads and assign barcoded reads to individual samples. Reads were kept if the sequence included a perfect match to the barcode and the V6 16S rRNA gene primers. Reads were clustered by 97% identity into operational taxonomic units (OTUs) using UClust v.3.0.617 [16]. OTUs that represented ≥1% of the reads in at least one sample were kept, while those that did not meet the cutoff were discarded. Taxonomic assignments for each OTU were made by the Ribosomal Database Project (RDP) SeqMatch tool [17]. From the top 20 matches to the RDP named isolates database, the full taxonomy was retained for matches with the highest S_ab score. For multiple top matches with equal scores, the highest common taxonomy was retained (for example, genus level if multiple species matched equally well). Since the maximum number of matches displayed per sequence is 20, the RDP taxonomic assignments were verified by BLAST against the Greengenes named isolates database with an output of 100 hits [18]. Taxonomy was assigned based on hits with the highest percentage identities and coverage. If multiple hits fulfilled this criterion, classification was re-assigned to a higher common taxonomy. In instances where the highest percentage identity/coverage yielded a single match, if this were <90% and the S_ab score from RDP was <0.7, taxonomy was assigned at the family level instead of at the genus level. A summary of each OTU classification and its sequence is shown in Additional file 1. The raw sequencing reads generated in this study have been deposited to the NCBI Short Read Archive (SRA) database [SRA:SRP041626].

#### Data analysis

Weighted UniFrac distances were calculated in QIIME [19] by using a phylogenetic tree of OTU sequences built with FastTree [20] and based on an OTU sequence alignment with MUSCLE [21]. The QIIME pipeline was also used to calculate Shannon’s diversity index (logarithms with base 2) and to generate principal coordinate analysis (PCoA) plots. Weighted UniFrac distances compare microbial profiles (presence/absence and abundance) between samples (i.e., beta-diversity) [22] while Shannon’s diversity index evaluates the microbial diversity within a sample (i.e., alpha diversity). The higher the Shannon’s diversity index, the more diverse a sample is and a value of zero indicates the presence of only one species [23]. PCoA plots allow one to visualize the UniFrac distance matrix and plot the values on a set of orthogonal axes that capture the greatest amount of variation between all samples tested. For beta-diversity analyses, the data set was rarified to the lowest read count/sample, which was 734 reads. A summary of clinical data, including total number of sequence reads per sample, is shown in Additional file 2. Barplots, boxplots and stripcharts were all generated in R [24].

#### Statistical analysis

The ALDEx R package version 2 [25] was used to compare genera between the non-treatment and chemotherapy treatment groups (as portrayed in the boxplots). Microbiome data represent proportional distributions and are thus not independent of each other. This means that a decrease in one organism will inevitably lead to a concomitant increase in another organism. For example, if a sample has two organisms A (50%) and B (50%) and A is completely killed by an antibiotic, the proportion of B in that sample will now be 100% even if its actual abundance has not changed. The ALDEx R package estimates the technical variation inherent in high-throughput sequencing by Monte-Carlo sampling from a Dirichlet distribution [26]. The Monte-Carlo replicates are transformed using the centered log-ratio transformation, which takes the logarithm of the Monte-Carlo estimates of organism abundances in each sample divided by the per-sample geometric mean organism abundance [27]. This transformation has several desirable properties that do not exist in proportional data, notably subcomposition coherence and linear sample independence. Data transformed in this way permit the use of standard statistical tests to determine significance. Values reported in this manuscript represent the expected values of 128 Dirichlet Monte-Carlo instances. A value of zero indicates that organism abundance is equal to the geometric mean abundance. Thus, organisms more abundant than the mean will have positive values and those less abundant than the mean will have negative values. Base 2 was used for the logarithm so differences between values represent fold changes. Statistical significance for these comparisons was determined by a Mann-Whitney U test with *P* < 0.05 and a false discovery rate (FDR) of < 0.1 using the q values output by the fdrtool R package [24].

The unpaired Student’s *t*-test was used to compare Shannon’s diversity index (*P* < 0.05).

### Sample preparation gas chromatography-mass spectrometry

To extract metabolites, 100 μl of milk were mixed with 400 μl pure methanol. Samples were vortexed for 15 s and centrifuged for 10 minutes at 9,000 *g*. Supernatants (200 μl) were transferred to gas chromatography-mass spectrometry (GC-MS) vials and 2.5 μl of ribitol solution (2 mg/ml) was added to each vial as an internal standard. Samples were dried to completeness using a SpeedVac. After drying, 100 μl of 2% methoxyamine•HCl in pyridine (MOX) was added to each sample for derivitization and samples were incubated at 50°C for 90 minutes. N-methyl-N-(trimethylsilyl) trifluoroacetamide (100 μl) was then added to each vial and incubated at 50°C for 30 minutes. Samples were transferred to microinserts before running on GC-MS (Agilent 7890A GC, 5975 inert MSD with triple axis detector, 30 m DB5-MS column with 10 m duraguard column). Samples were run using 1 μl injections on scan mode and a solvent delay of 10 minutes. Run time was 60 minutes per sample. Each sample was run twice non-consecutively to ensure consistency throughout the sequence.

### GC-MS data analysis

Chromatogram files were converted to ELU format using the AMDIS Mass Spectrometry software [28]. Chromatograms were aligned and abundance of metabolites calculated using the Spectconnect software [29] with the support threshold set to low. In order to determine if differences between week 0 and chemotherapy (weeks 2 to 16) existed, principle component analysis (PCA) was conducted in SIMCA (Umetrics, San Jose, CA, USA) using the relative abundance matrix output from Spectconnect. Data were mean centered and pareto scaled prior to PCA. Independent unpaired *t*-tests with Bonferroni correction were calculated in Excel to determine metabolites that were significantly altered by chemotherapy (*P* < 0.05). Compounds that also contributed to the separation of week 0 from chemotherapy samples according to the PCA loadings plot (compounds in bottom left quadrant) were chosen for further investigation.

## Findings

**Figure 2:**
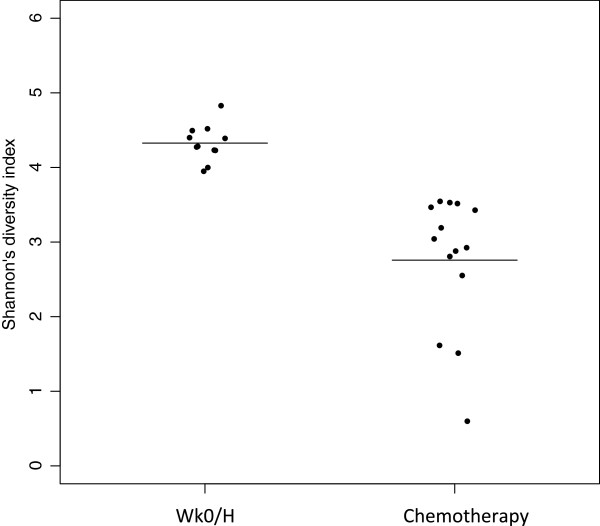
**Changes in bacterial diversity as a result of chemotherapy.** Bacterial diversity within a sample (i.e. alpha diversity) was measured by calculating Shannon’s diversity index. Each point on the graph represents a subject with the line representing the mean for all samples within a group. The higher the index the greater the bacterial diversity found within a sample. The mean of the ‘Wk0/H’ group (week 0 and healthy samples) was 4.3, and that of the chemotherapy group (weeks 2 to 16) was 2.8. Groups were statistically different from each other as measured by unpaired Student’s *t*-test (*P* < 0.05).

**Figure 3:**
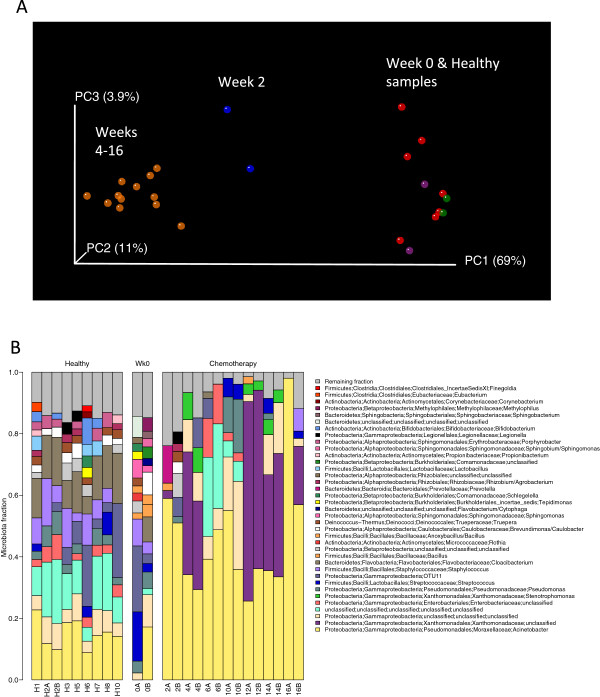
**16S rRNA sequencing analysis of bacteria in human milk.** Milk samples were collected from a lactating woman undergoing chemotherapy as described in Figure 1 as well as from eight healthy lactating women. **(A)** Weighted UniFrac PCoA plot. Each milk sample, represented by a coloured circle, is plotted on this three-dimensional, three-axis plane representing 84% of the variation observed between all samples. Samples that cluster together are similar in biota composition and abundance. Orange circles represent samples collected from weeks 4 to 16 of chemotherapy, blue circles represent samples collected at week 2 of chemotherapy, purple circles represent samples collected at week 0, red circles represent milk samples from healthy lactating women (only one time point) and green circles represent milk samples from a healthy lactating women collected 4-months apart. As shown by the plot, there were three distinct groups: (i) week 0 samples and healthy milk samples; (ii) week 2 of chemotherapy; and (iii) weeks 4 to 16 of chemotherapy. Data were rarified to 735 reads/sample. **(B)** Barplot showing the relative abundances of different genera in each sample. Each bar represents a subject and each coloured box a different genus. The height of the coloured boxes represents the relative abundance of that genus within the sample. Genera that were less than 2% abundant in a given sample were placed in the ‘Remaining fraction’ at the top of the graph (grey boxes).

**Figure 4:**
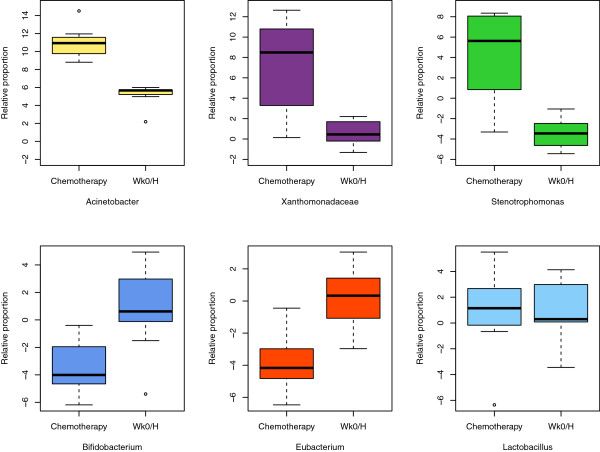
**Comparison of relative proportions of bacterial taxa between treatments.** Boxplots comparing six bacterial taxa between samples collected during chemotherapy (weeks 4 to 16) and those without treatment (week 0 and healthy samples (Wk0/H)). The box signifies the 75% (upper) and 25% (lower) quartiles and thus shows where 50% of the samples lie. The black line inside the box represents the median. The whiskers represent the lowest datum still within 1.5 interquartile range (IQR) of the lower quartile and the highest datum still within 1.5 IQR of the upper quartile. Outliers are shown with open circles. The value ‘0’ represents the geometric mean abundance; thus, values above 0 are more abundant and values less than 0 are less abundant than the geometric mean. Significant differences were observed between the two groups for all taxa graphed (Mann-Whitney U test *P* < 0.05, FDR <0.1).

Chemotherapy affected both bacterial diversity and bacterial profiles in human milk. Bacterial diversity within samples was lower in milk collected throughout chemotherapy compared with milk samples collected at week 0 and from healthy lactating women (Figure 2). Bacterial profiles at week 0 were similar to those from healthy women, although this changed within 2 weeks of treatment (Figure 3A). Samples collected at weeks 4 to 16 shared similar profiles and differed from week 2 and from week 0/healthy samples (Figure 3A). These differences were not due to natural changes over time, as the bacterial community in two milk samples analyzed from a healthy subject did not change over a 4-month period (Figure 3A, green samples). The bar plot in Figure 3B shows the bacterial communities present in these samples with a striking increase in abundances of *Acinetobacter* and *Xanthomonadaceae* in milk collected during chemotherapy. A comparison of relative abundances of *Acinetobacter*, *Xanthomonadaceae* and *Stenotrophomonas* (a genus belonging to the Xanthomonadaceae family) between the chemotherapy (weeks 4 to 16) and non-treatment (week 0 and healthy samples) groups is displayed in Figure 4 and were significantly higher during chemotherapy. We also examined the differences between three bacteria believed to confer beneficial health effects to the infant, *Bifidobacterium*, *Eubacterium* and *Lactobacillus*. The first two were significantly decreased during chemotherapy whereas no differences were observed for *Lactobacillus* (Figure 4). Overall, a total of 22 out of the 49 genera identified were differentially abundant between the two groups (Additional file 3). While the core microbiome (that is, taxa that were present in 100% of the samples) was somewhat similar between the two groups, it is interesting to note that *Stenotrophomonas* was present in every chemotherapy sample and *Lactobacillus* and *Eubacterium* were present in every healthy and week 0 sample (Additional file 4).

**Figure 5:**
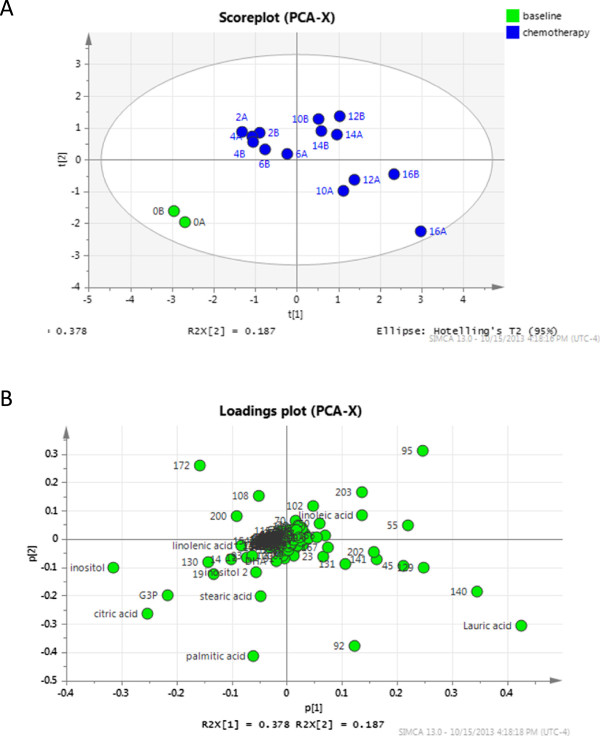
**Principle component analysis of metabolites in breast milk at week 0 and during chemotherapy. (A)** Scoreplot displaying the distribution of samples based on metabolites alone, where the distance between samples represents how similar the metabolome of those samples are. Each point represents the average of two technical replicates. **(B)** Loadings plot. Each point represents a metabolite. Metabolites present in a given quadrant of the loadings plot are present in highest abundance in samples present in the same quadrant of the scoreplot (A).

The metabolic profile also changed as a result of chemotherapy (Figure 5) and was similar between weeks 2 and 16, but different to that observed at week 0. A total of 226 metabolites were detected by our GC-MS method, 12 of which were significantly different between the week 0 and chemotherapy (weeks 2 to 16) groups (Table 1). Additional file 5 shows the relative abundances of all metabolites detected in milk and stripcharts in Additional file 6 show the distribution of key metabolites detected between the two groups.

**Table 1:** Metabolites significantly altered by chemotherapy

**Metabolite**	**Elevated in**	**Bonferroni corrected**
		***P *** **value**
Unknown PUFA	Week 0	1.81E-07
DHA^a^	Week 0	0.000304
Arabinose	Chemotherapy	0.000456
Threitol	Chemotherapy	0.001342
Unknown	Chemotherapy	0.002685
Unknown	Chemotherapy	0.002768
Decanoic acid	Chemotherapy	0.008458
Myristic acid	Chemotherapy	0.008727
1-Monopalmitin	Chemotherapy	0.009143
Butanal	Chemotherapy	0.012356
Unknown	Chemotherapy	0.017961
Inositol^a^	Week 0	0.037225

^a^Metabolite identity confirmed by authentic standards. Chemotherapy refers to samples collected from weeks 2 to 16. Significant differences were determined using the unpaired Student's *t*-test, *P* < 0.05. DHA, docosahexaenoic acid, PUFA, polyunsaturated fatty acid.

## Discussion

This study shows that a course of chemotherapy has significant effects on bacterial and metabolic profiles in human milk, moving away from those of healthy lactating women. Of note, the subject did not report any additional drug use, antibiotics, illness or major changes in diet over the course of the study.

The consequences of decreased bacterial diversity in human milk and the implications on the child are still unknown; however, the decreased milk diversity could impact intestinal diversity and it has been shown that low intestinal diversity in the first weeks of life is associated with necrotizing enterocolitis [30] and an increased risk of allergy and atopy in school-age children [31]. Lower intestinal diversity has also been observed in children with type 1 diabetes compared to age-matched controls [32].

In addition to overall changes in microbial profiles, we observed a significant decrease in the relative abundance of *Bifidobacterium* in the chemotherapy group compared to the non-treatment group. *Bifidobacterium* is the predominant organism in the gut of breastfed infants, attributed to its ability to metabolize the human milk oligosaccharides present in large amounts in milk [33,34]. Maternal levels can also impact gut *Bifidobacterium* abundance, with low levels in milk correlating with low levels in the neonatal gut [35]. The potential consequences of decreased numbers of *Bifidobacterium* being passed on from the mother to the neonate could be an increased risk of asthma or obesity later in life. High levels of *Bacteroides* have been reported in the gut of infants with low levels of *Bifidobacterium*[36] and early colonization with high *Bacteroides* counts has been associated with an increased risk of developing asthma and obesity [37-39]. In addition, depleted levels of *Bifidobacterium* have been shown to promote colonization of opportunistic pathogens such as *Klebsiella* and *Citrobacter*[36].

*Staphylococcus*, including coagulase negative species, are one of the predominant organisms in human milk [40-42], and were also significantly reduced as a result of chemotherapy. It has been shown that numerous human milk isolates of *Staphylococcus epidermidis* can inhibit the growth of *Staphylococcus aureus*[43], the main causative agent of mastitis, which is a painful inflammatory condition of the breast that often leads to premature cessation of breastfeeding in many women. While we were not able to identify the *Staphylococcus* in our samples down to the species level with 16S rRNA gene sequencing, culture analysis on mannitol salt agar plates did show that the *Staphylococcus* isolates were not *S. aureus* and the select few that were tested were coagulase negative. This reduction of *Staphylococcus* (likely coagulase negative species) as a result of chemotherapy could make lactating women more prone to infections, affecting both themselves and their infants. Like *Bifidobacterium*, *Staphylococcus* is passed from the milk to the neonate, with higher numbers in the intestine of breastfed compared to formula-fed infants [1]. Interestingly, a metagenome analysis revealed the presence of immunosuppressive motifs in bacterial DNA from human milk, with the majority of these belonging to *Staphylococcus*[41]. The exposure of the neonate to this DNA, either ingested from the milk or through live bacteria that have released their DNA once in the gut, could help to regulate the infant’s immune response against a variety of innocuous bacterial, environmental and food antigens.

The utilization of bacterial products by other bacteria is termed metabolic cross-feeding and plays an important role in bacterial selection. For example, the byproducts of bacterial metabolism, such as lactate and acetate production, are utilized as an energy source by many butyrate-producing bacteria [44-47] such as *Eubacterium*, which was decreased in our chemotherapy group. Butyrate is important for health, as it reduces inflammation and metabolic diseases, promotes colonic repair and protects against colon cancer [48,49]. On the other hand, some pathogens persist and cause disease only in the presence of certain commensal bacteria [50], likely due to the metabolites produced. Thus, changes in bacterial communities in human milk will inevitably alter the metabolic milieu, selecting for bacteria able to utilize those metabolites. As a result, potential shifts from a healthy and balanced intestinal microbiota can occur, having important consequences on health.

Docosahexaenoic acid (DHA), inositol and an unknown polyunsaturated fatty acid were among the 12 metabolites that differed between week 0 and weeks 2 to 16, with reduced levels detected during chemotherapy (weeks 2 to 16). DHA is the most abundant long chain polyunsaturated fatty acid in the brain, retina and nerve cells and is supplied mainly through breast milk [51]. DHA deficiencies lead to reduced brain, eye and neuronal development [51] and it has been observed that breastfed infants have better visual acuity and neuronal development compared to those fed formula [52]. Reduced levels of DHA and alpha-linolenic acid (a precursor of DHA) have been reported in milk of mothers with atopic children compared to milk from mothers with non-atopic children [53,54]. Another principal metabolite in the neonatal brain is inositol, which is important for osmoregulation, cellular nutrition and detoxification [55]. Palmitic acid levels were also reduced during chemotherapy, though the results were not significant. Palmitic acid is the most abundant lipid in human milk and has been shown to increase bone strength in infants [56] and limit intestinal damage and pro-inflammatory immune responses in mice [57]. While changes in metabolite concentrations do occur over the course of lactation, especially between colostrum and mature milk, no changes in the above metabolites over the course of the first year of life have been observed in mature milk [58-61]. Due to the high variability in milk metabolites between individuals, we did not have enough samples from healthy women to make substantive claims as to how the Hodgkin’s patient compared, although there were no obvious differences in the metabolic profiles of control samples taken at early compared to later stages of lactation.

We recognize that the main limitation of the study is its single case study content of a patient undergoing chemotherapy. However, the findings were revealing. Of note, even with the disease, her milk microbiota before treatment was similar to that of healthy lactating women and only after intervention did the microbiota patterns alter. It has been reported by Hunt *et al.*[40] that the milk microbiota of a specific individual is stable over time, consistent with our study from the healthy sample collected 4 months apart. We believe that the changes in the microbiota are a result of therapeutic agents and not specific to just one patient.

## Conclusions

Bacterial and metabolic compositions in human milk, so critical for immunity and infant development, can change significantly after maternal exposure to chemotherapeutic agents. Further larger cohort studies are warranted to examine microbiota and metabolomic changes associated with chemotherapy and other medications prescribed to lactating mothers and the consequences for the microbiome, the metabolome and long-term health of infants.

## Supporting data

The data set supporting the results of this article are included in Additional files 1, 2, 3, 4, 5 and 6.

## Abbreviations

DHA: docosahexaenoic acid; FDR: false discovery rate; GC-MS: gas chromatography-mass spectrometry; OTU: operational taxonomic unit; PCA: principal component analysis; PCR: polymerase chain reaction; PCoA: principal coordinate analysis; RDP: Ribosomal Database Project.

## Competing interests

The authors declare that they have no competing interests.

## Authors’ contributions

CU designed the study, recruited subjects, collected and prepared the samples for microbial analysis, analyzed 16S rRNA sequencing data and wrote the manuscript. AM performed GC-MS analysis on the milk samples and reviewed the manuscript. MA helped with study design and recruitment and reviewed the manuscript. JB provided input into study design and reviewed the manuscript. MS contributed substantially to the design and acquisition of the metabolomic data, analysis and manuscript review. GG provided co-supervision, instruction and input into microbiome acquisition and analysis and manuscript review. GR conceptualized the study, helped with study design and manuscript writing, supervised data collection and analysis and provided financial support. All authors read and approved the final manuscript.

## Supplementary Material

Additional file 1: Table S1 Summary of taxonomic results and full-length V6 16S rRNA sequence of each OTU. Click here for file

Additional file 2: Table S2 Summary of clinical data. Click here for file

Additional file 3: Table S3 Comparison of relative abundances of different genera detected in milk between the chemotherapy and non-treatment groups. Values in the second and third columns represent the base 2 logarithm of the median abundance in all samples within a group (that is, Wk0/H samples (non-treatment group) or Wk4-16 samples (chemotherapy group) relative to the geometric mean abundance, which has a value of 0. Thus, positive values are higher than the geometric mean and are thus more abundant than negative values, which are lower than the geometric mean. Significant differences were based on FDR values of <0.1. Out of the 49 genera identified, 22 were significantly different between the two groups. Click here for file

Additional file 4: Table S4 Taxa present in every sample within a group. Click here for file

Additional file 5: Table S5 Relative abundances of metabolites identified in breast milk by GC-MS. Relative abundance values were constructed by the Spectconnect program, which assigns the sample with the greatest concentration (peak area) of each metabolite an arbitrary value. All other samples are then made proportional to this value according to peak area. Values represent an average of two technical replicates. *Metabolite identity confirmed by authentic standards. The metabolites referred to with numbers in the first column represent un-annotated metabolites. The *P* values between the week 0 and chemotherapy groups were calculated using an unpaired *t*-test with Bonferroni correction. Click here for file

Additional file 6: Figure S1 Comparison of relative abundance of metabolites in milk between week 0 and chemotherapy. The relative abundances of DHA, inositol and palmitic acid are shown above, with each point on the graph representing a different milk sample. DHA and inositol were significantly decreased by chemotherapy (unpaired *t*-test with Bonferroni correction, *P* < 0.05). While palmitic acid was also decreased by chemotherapy, these results were not statistically significant. Values represent an average of two technical replicates. Click here for file
