# Supplementary material for: Effect of chemotherapy on the microbiota and metabolome of human milk, a case report
Source: Microbiome. 2014 Jul 11;2:24. doi: 10.1186/2049-2618-2-24 (PMC4109383; doi:10.1186/2049-2618-2-24)
Supplement: Additional file 3: Table S3 — Comparison of relative abundances of different genera detected in milk between the chemotherapy and non-treatment groups. Values in the second and third columns represent the base 2 logarithm of the median abundance in all samples within a group (that is, Wk0/H samples (non-treatment group) or Wk4-16 samples (chemotherapy group) relative to the geometric mean abundance, which has a value of 0. Thus, positive values are higher than the geometric mean and are thus more abundant than negative values, which are lower than the geometric mean. Significant differences were based on FDR values of <0.1. Out of the 49 genera identified, 22 were significantly different between the two groups. [file 2049-2618-2-24-S3.doc]

| **Taxa** | **Wk0/H** | **Chemo(Wk4-16)** | **p-value** | **FDR value** | **Higher in** |
| --- | --- | --- | --- | --- | --- |
| *Acinetobacter* | 5.542 | 10.88 | 0 | 0 | Chemo |
| *Gammaproteobacteria* | 4.372 | 7.901 | 0 | 0.001 | Chemo |
| *Stenotrophomonas* | -3.181 | 5.751 | 0 | 0.002 | Chemo |
| *Xanthomonadaceae* | 0.605 | 8.547 | 0.001 | 0.003 | Chemo |
| *Cloacibacterium* | 5.301 | 1.981 | 0.001 | 0.003 | Wk0/H |
| *Rhizobiales* | -1.947 | 2.541 | 0.001 | 0.003 | Chemo |
| *Porphyrobacter* | 2.763 | 0.259 | 0.001 | 0.005 | Wk0/H |
| *Thermus* | -2.361 | 1.733 | 0.002 | 0.006 | Chemo |
| *Streptococcus* | 2.313 | 4.696 | 0.001 | 0.007 | Chemo |
| *Flavobacterium/Cytophaga* | 0.169 | 3.062 | 0.002 | 0.007 | Chemo |
| *Bifidobacterium* | 0.74 | -3.51 | 0.002 | 0.008 | Wk0/H |
| *Eubacterium* | 0.224 | -3.607 | 0.003 | 0.01 | Wk0/H |
| *Rothia* | -1.221 | 2.325 | 0.002 | 0.011 | Chemo |
| *Brevundimonas/Caulobacter* | 3.105 | -0.117 | 0.003 | 0.013 | Wk0/H |
| *Microbacteriaceae* | -1.449 | 1.322 | 0.006 | 0.022 | Chemo |
| *Staphylococcus* | 4.445 | 2.306 | 0.008 | 0.028 | Wk0/H |
| *Anoxybacillus/Bacillus* | 1.616 | 3.53 | 0.007 | 0.029 | Chemo |
| *Bacillus* | 0.243 | 2.623 | 0.008 | 0.03 | Chemo |
| *Gammaproteobacteria* OTU11 | 3.729 | 0.3 | 0.017 | 0.052 | Wk0/H |
| *Petrobacter* | 0.184 | -2.054 | 0.025 | 0.076 | Wk0/H |
| *Truepera* | 2.9 | -0.271 | 0.026 | 0.081 | Wk0/H |
| *Betaproteobacteria* | 2.579 | -0.119 | 0.026 | 0.093 | Wk0/H |
| *Enterobacteriaceae* | 3.204 | 5.026 | 0.036 | 0.123 | N/A |
| *Sphingomonas* | 0.939 | 2.103 | 0.047 | 0.139 | N/A |
| *Faecalibacterium* | -0.361 | -2.055 | 0.055 | 0.156 | N/A |
| *Tepidimonas* | 0.662 | -2.512 | 0.048 | 0.159 | N/A |
| *Comamonadaceae* | 1.21 | 2.257 | 0.064 | 0.195 | N/A |
| *Schlegelella* | 1.058 | -1.364 | 0.067 | 0.251 | N/A |
| *Pseudomonas* | 3.494 | 4.771 | 0.098 | 0.359 | N/A |
| *Finegoldia* | -2.234 | -4.213 | 0.186 | 0.42 | N/A |
| *Bacteroidetes* | -3.188 | -5.058 | 0.248 | 0.463 | N/A |
| *Prevotella* | -3.061 | -5.022 | 0.227 | 0.467 | N/A |
| *Legionella* | -2.686 | -4.538 | 0.271 | 0.554 | N/A |
| *Corynebacterium* | -1.075 | -2.789 | 0.235 | 0.569 | N/A |
| *Sphingobium/Sphingomonas* | 1.136 | -1.244 | 0.21 | 0.584 | N/A |
| *Porphyromonas* | -3.614 | -5.246 | 0.306 | 0.611 | N/A |
| *Rhizobium/Agrobacterium* | 1.212 | -0.374 | 0.23 | 0.632 | N/A |
| *Ruminococcus* | -3.541 | -4.725 | 0.36 | 0.642 | N/A |
| *Flavobacteriaceae* | -3.811 | -5.116 | 0.346 | 0.642 | N/A |
| *Gemella* | -2.353 | -0.957 | 0.375 | 0.766 | N/A |
| *Peredibacter* | -2.9 | -4.141 | 0.423 | 0.768 | N/A |
| *Sphingobacterium* | -2.283 | -3.517 | 0.476 | 0.781 | N/A |
| *Atopobium* | -3.526 | -3.942 | 0.552 | 0.867 | N/A |
| *Methylophilus* | -3.242 | -1.833 | 0.517 | 0.879 | N/A |
| *Brevundimonas* | 1.479 | 0.797 | 0.483 | 0.918 | N/A |
| *Gardnerella* | -2.188 | -2.203 | 0.637 | 0.933 | N/A |
| *Propionibacterium* | 2.103 | 2.182 | 0.721 | 0.954 | N/A |
| *Lactobacillus* | 0.891 | 1.273 | 0.837 | 0.993 | N/A |
| unclassified *Bacteria* | 5.004 | 4.489 | 0.874 | 1 | N/A |
