# Supplementary figures and images for: Effect of chemotherapy on the microbiota and metabolome of human milk, a case report
Source: Microbiome. 2014 Jul 11;2:24. doi: 10.1186/2049-2618-2-24 (PMC4109383; doi:10.1186/2049-2618-2-24)

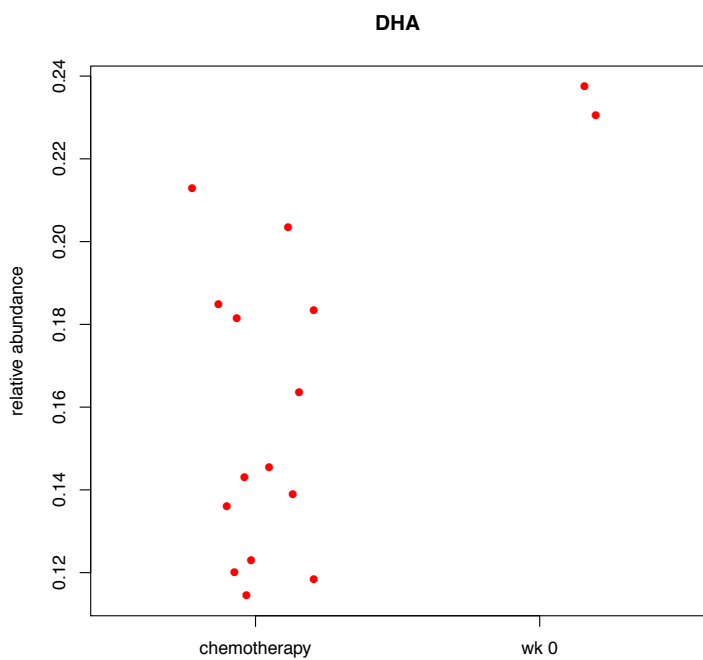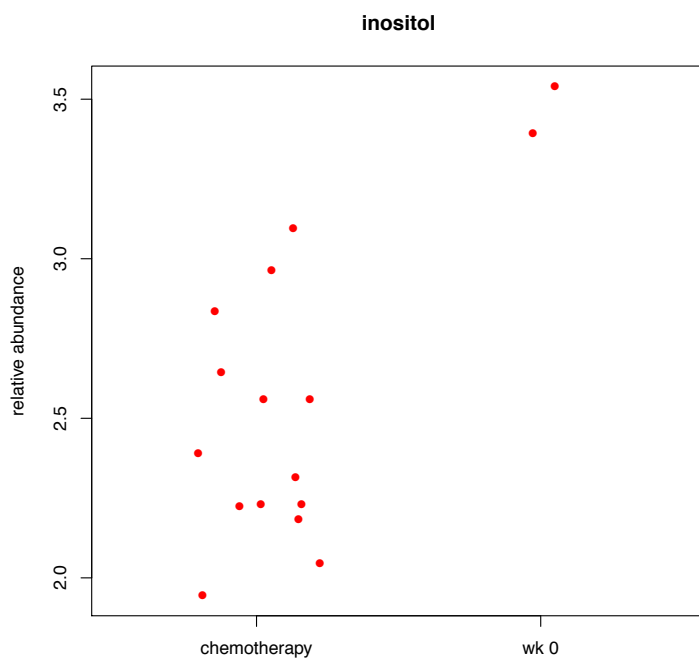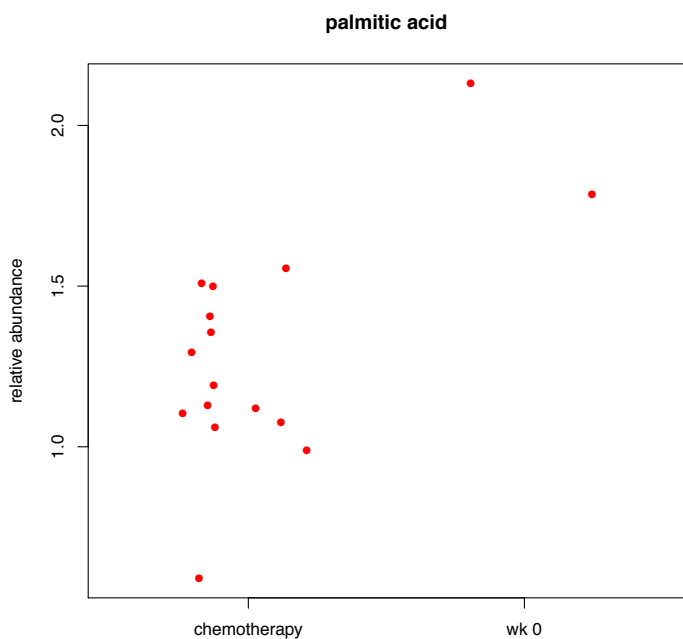

Supplement: Additional file 6: Figure S1 — Comparison of relative abundance of metabolites in milk between week 0 and chemotherapy. The relative abundances of DHA, inositol and palmitic acid are shown above, with each point on the graph representing a different milk sample. DHA and inositol were significantly decreased by chemotherapy (unpaired t-test with Bonferroni correction, P < 0.05). While palmitic acid was also decreased by chemotherapy, these results were not statistically significant. Values represent an average of two technical replicates. [file 2049-2618-2-24-S6.pdf]
